# Three-input gate logic circuits on chemically assembled single-electron transistors with organic and inorganic hybrid passivation layers

**DOI:** 10.1080/14686996.2017.1320190

**Published:** 2017-05-31

**Authors:** Yutaka Majima, Guillaume Hackenberger, Yasuo Azuma, Shinya Kano, Kosuke Matsuzaki, Tomofumi Susaki, Masanori Sakamoto, Toshiharu Teranishi

**Affiliations:** ^a^ Laboratory for Materials and Structures, Tokyo Institute of Technology, Yokohama, Japan.; ^b^ Materials Research Center for Element Strategy, Tokyo Institute of Technology, Yokohama, Japan.; ^c^ Institute for Chemical Research, Uji, Kyoto, Japan.

**Keywords:** Single-electron transistor, passivation, aluminum oxide, chemical assembly, logic circuit, nanoparticle, 40 Optical, magnetic and electronic device materials, 201 Electronics / Semiconductor / TCOs, 101 Self-assembly / Self-organized materials, 202 Dielectrics / Piezoelectrics / Insulators, 106 Metallic materials

## Abstract

Single-electron transistors (SETs) are sub-10-nm scale electronic devices based on conductive Coulomb islands sandwiched between double-barrier tunneling barriers. Chemically assembled SETs with alkanethiol-protected Au nanoparticles show highly stable Coulomb diamonds and two-input logic operations. The combination of bottom-up and top-down processes used to form the passivation layer is vital for realizing multi-gate chemically assembled SET circuits, as this combination enables us to connect conventional complementary metal oxide semiconductor (CMOS) technologies via planar processes. Here, three-input gate exclusive-OR (XOR) logic operations are demonstrated in passivated chemically assembled SETs. The passivation layer is a hybrid bilayer of self-assembled monolayers (SAMs) and pulsed laser deposited (PLD) aluminum oxide (AlO${}_{x}$), and top-gate electrodes were prepared on the hybrid passivation layers. Top and two-side-gated SETs showed clear Coulomb oscillation and diamonds for each of the three available gates, and three-input gate XOR logic operation was clearly demonstrated. These results show the potential of chemically assembled SETs to work as logic devices with multi-gate inputs using organic and inorganic hybrid passivation layers.

## Graphical abstract

**Figure:**
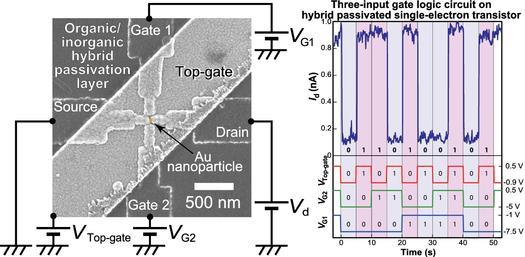

## 1. Introduction

Novel sub-10-nm scale transistors are required for the realization of next-generation electronics, since a widely accepted design rule for semiconductor devices is that they must shrink over time, down to 5 nm within the next 10 years based on current projections [1]. Nowadays, silicon-based multi-gate field effect transistors (FETs) such as Fin-FET are used in practical applications, but FETs must overcome their short-channel effects and reduce their leakage currents in order to minimize the power consumption of their electronic circuits [1]. Fabrication and functionalization of novel sub-10-nm scale electronic devices based on new materials are key issues for development in the next 10 years.

Single-electron electronics have received increasing attention in recent years, because this field may lead to a new generation of faster processors with low power consumption [2,3]. Single-electron transistors (SETs), one candidate in the realm of sub-10-nm scale devices, are operated by Coulomb blockade effects with conductive Coulomb islands. As such, the total numberof SETs required for logic operations can be decreased by utilizing the multi-gate structure, in which the linear combination of gate voltages operates the phase shift of the Coulomb oscillation [2,3]. Logic operations based on single electronics with various materials such as GaAs and CdS nanowires have been reported [4–8].

Despite numerous efforts to reduce the size of FETs, several transistors are usually required to realize certain logic functions [3]. However, combining several SETs allows for logic operation with low-energy circuits [9–19]. Logic operations with single carbon nanotube SETs have also been demonstrated previously [20]. In both of these cases, two-gate inputs were used to demonstrate these logic properties.

Recently, we have reported chemically assembled SETs formed using top-down and bottom-up technologies. Au nanogap electrodes with double side-gate electrodes were simultaneously fabricated using electron-beam lithography (EBL) and electroless gold plating (ELGP) [21–25]. The narrowing of the gap separation between the electrodes is made possible by electroless Au plating and stops at 3.0 nm, owing to a self-termination mechanism [26–28]. The surfaces of the self-terminated nanogap electrodes are covered by alkanethiol and alkanedithiol mixed self-assembled monolayers (SAMs). Double-gate SETs were fabricated by anchoring of chemically synthesized colloidal Au nanoparticles using alkanedithiol [22,29,30]. The fabricated SETs show highly stable and reproducible Coulomb diamonds under applied voltages at the source and gate electrodes [31]. The sole SET also exhibited all usual types of two-input logic operations (XOR, XNOR, NAND, OR, NOR, and AND) with an on/off ratio of 10${}^{2}$, indicating the potential of chemical assembly to yield highly stable SETs exhibiting all logic operations [31].

Hybrid insulators of SAMs and inorganic bilayers show good gate insulator properties when used in organic thin-film transistors [32–35]. We have also demonstrated chemically assembled transistors made by utilizing a hybrid passivation bilayer [36]. SiN${}_{x}$ was prepared by catalytic chemical vapor deposition (CAT-CVD) at 338 K onto alkanethiol and alkanedithiol mixed SAMs to form a hybrid passivation bilayer, and a top-gate electrode was added on the Au nanoparticle via an EBL overlay method at a process temperature of 423 K [36,37]. Even with Coulomb islands of colloidal Au nanoparticles covered by organic alkanethiol molecules, the hybrid passivated SETs showed clear and stable Coulomb diamonds with applied top-gate voltage [36].

Here, we report three-input gate logic operations on a single chemically assembled SET with hybrid passivated bilayers based on mixed SAMs and pulsed laser deposited (PLD) aluminum oxide (AlO${}_{x}$). The top-gate electrode was prepared on a high-k material of an AlO${}_{x}$ passivation layer. The chemically assembled SET consists of a Au nanoparticle Coulomb island, top-gate electrodes together with two side-gate electrodes (with clear Coulomb blockade behavior), and Coulomb diamonds for all three-input gates at 9 K. XOR logic operations with the three-input gates are demonstrated by applying simultaneous pulse gate voltages. These results extend the possibility of combining top-down and bottom-up adoption technologies for the fabrication of chemically assembled SET logic circuits, in which organic alkanethiols and inorganic materials are used as hybrid insulators.

## 2. Experimental details

The chemically assembled SET used in this paper was fabricated through a combination of top-down and bottom-up methods based on previous studies [21–23]. Electrode patterns were designed by EBL (Elionix, ELS-7500EX, Hachioji, Japan) on a SiO${}_{2}$ (300 nm)/Si substrate, and a Ti/Au layer was evaporated onto the substrate under high vacuum conditions. At this point, the average gap separation is approximately 25 nm. Probing electrodes were then added by photolithography, and an iodine electroless Au plating solution was used to reduce the size of the nanogaps to less than 5 nm via a self-termination mechanism [26–28]. Chemically synthesized decanethiol-protected Au nanoparticles with a core diameter of 6.2 ± 0.8 nm were then chemisorbed between the nanogap electrodes using decanedithiol and octanethiol mixed self-assembled monolayers (SAMs) on the nanogap electrodes [38–43].

**Figure 1.**
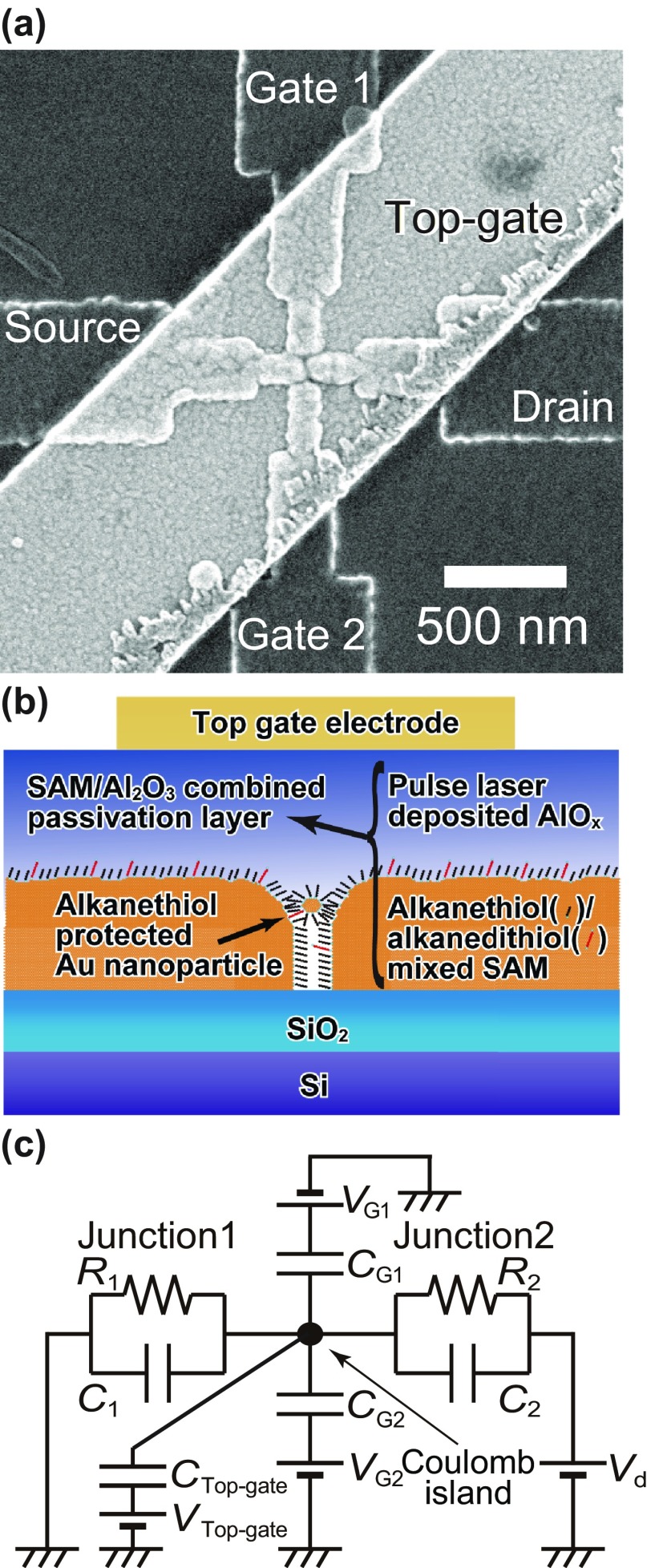
(a) SEM top-view image of hybrid passivated chemically assembled SET with two side-gates (1 and 2), and one top-gate. (b) Cross-sectional image of the hybrid SAM and AlO${}_{x}$-passivated chemically assembled SET with a gold top-gate electrode. (c) Equivalent circuit of the SET with a Coulomb island comprised of a 6.2-nm-diameter chemically synthesized Au nanoparticle, two side-gate electrodes (1 and 2), and one top-gate electrode. The SET parameters are as follows: $R_{1}$, the tunneling resistance between the source electrode and the Au core of the nanoparticle; $R_{2}$, the tunneling resistance between the drain electrode and the Au core; $C_{1}$, the capacitance between the source electrode and the Au core; $C_{2}$, the capacitance between the drain electrode and the Au core; $C_{G}1$, the side-gate 1 capacitance; $C_{G}2$, the side-gate 2 capacitance; $C_{t}op-gate$, the top-gate capacitance.

In this study, we introduce alkanethiol/alkanedithiol mixed SAM and inorganic AlO${}_{x}$ as hybrid passivation materials. The top view scanning electron microscopy (SEM) image of the three-input gate SET is shown in Figure 1(a). An AlO${}_{x}$ layer (50 nm) was deposited at room temperature by PLD [44]. Finally, Ti/Au (30 nm/70 nm) top-gate electrodes were added using an EBL-based overlay method with a poly(methyl methacrylate) resist layer applied with pre-baking at 423 K for 10 min [36,45]. As shown in Figure 1(a), the top-gate electrodes fully cover the nanogap area, where Coulomb islands of chemically anchored colloidal Au nanoparticles exist beneath the hybrid passivation layer. We can clearly observe the source and drain electrodes along with the two side-gates (gate 1 and gate 2) and the top-gate. A cross-sectional schematic diagram of the final SET is shown in Figure 1(b).

The electron transport properties of the SET were measured using a mechanical refrigerator-type probe (Nagase, GRAIL10-LOGOS01S, Nihonbashi, Japan) and asemiconductor device analyzer (B1500, Agilent, Santa Rosa, CA, USA). The measurement temperature was 9 K. All measurements were conducted under the same conditions as in previous studies [21–23]. The vacuumpressure in the measurement chamber was approximately $10^{-6}$ Pa. The stability diagram of the two-dimensional d$I_{d}$/d$V_{d}$ plot was acquired by differentiating the $I_{d}$–$V_{d}$ curve numerically. Finally, the logic operation was demonstrated by applying simultaneous three-input gate pulse voltages and observing the $I_{d}$ current response.

## 3. Results and discussion

**Figure 2.**
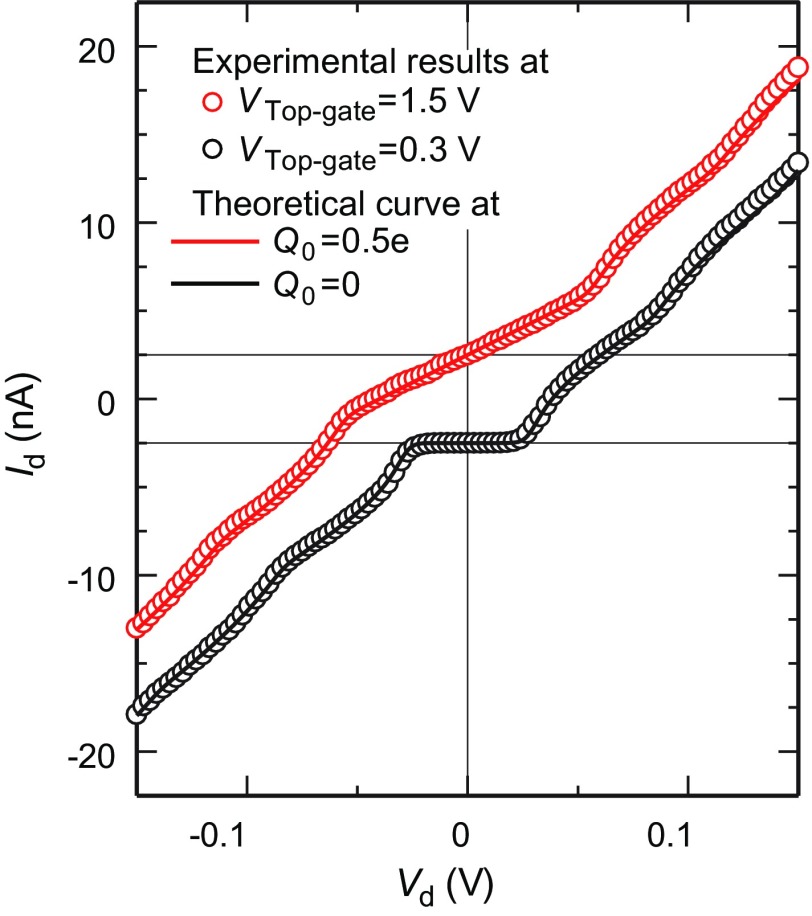
Experimental results of $I_{d}-V_{d}$ curves for different gate voltages of $V_{G}1$=0, $V_{G}2$=0 and $V_{t}op-gate$=1.5 V (red open circle), and $V_{G}1$=0, $V_{G}2$=0 and $V_{t}op-gate$=0.3 V (black open circle) at 9 K. Theoretical $I_{d}-V_{d}$ curves obtained from the orthodox model at different fractional charges $Q_{0}$ of 0.5*e* (red solid line) and 0 (black solid line). The other evaluated SET parameters of $R_{1}$, $R_{2}$, $C_{1}$ and $C_{2}$ are 1.6 MΩ, 6.5 MΩ, 2.9 aF, and 2.9 aF, respectively. The coordinate origins are shifted to 2.5 nA (red open circle and red solid line) and –2.5 nA (black open circle and black solid line) for clarity.

Drain current vs. drain voltage ($I_{d}$–$V_{d}$) characteristics of chemically assembled SETs with both the hybrid passivation due to SAMs and AlO${}_{x}$, and top-gate electrodes under top-gate voltages ($V_{t}op-gate$) of 0.3 and 1.5 V, are shown in Figure 2. Clear Coulomb staircases were observed at 9 K. The theoretical curves at offset charges $Q_{0}$ of 0 and 0.5*e* (*e*: unit charge) were fitted to the experimental results using the orthodox model [46,47]. The evaluated SET parameters of $R_{1}$, $R_{2}$, $C_{1}$ and $C_{2}$ are 1.6 MΩ, 6.5 MΩ, 2.9 aF, and 2.9 aF, respectively (Figure 1(c)). Two theoretical curves coincide well with the experimental Coulomb staircases, indicating that the hybrid SAM and AlO${}_{x}$ passivated SET still functioned after the PLD and EBL processes for the passivation and top-gate electrode, respectively, during which the sample was pre-baked at 423 K for 10 min within the EBL process.

**Figure 3.**
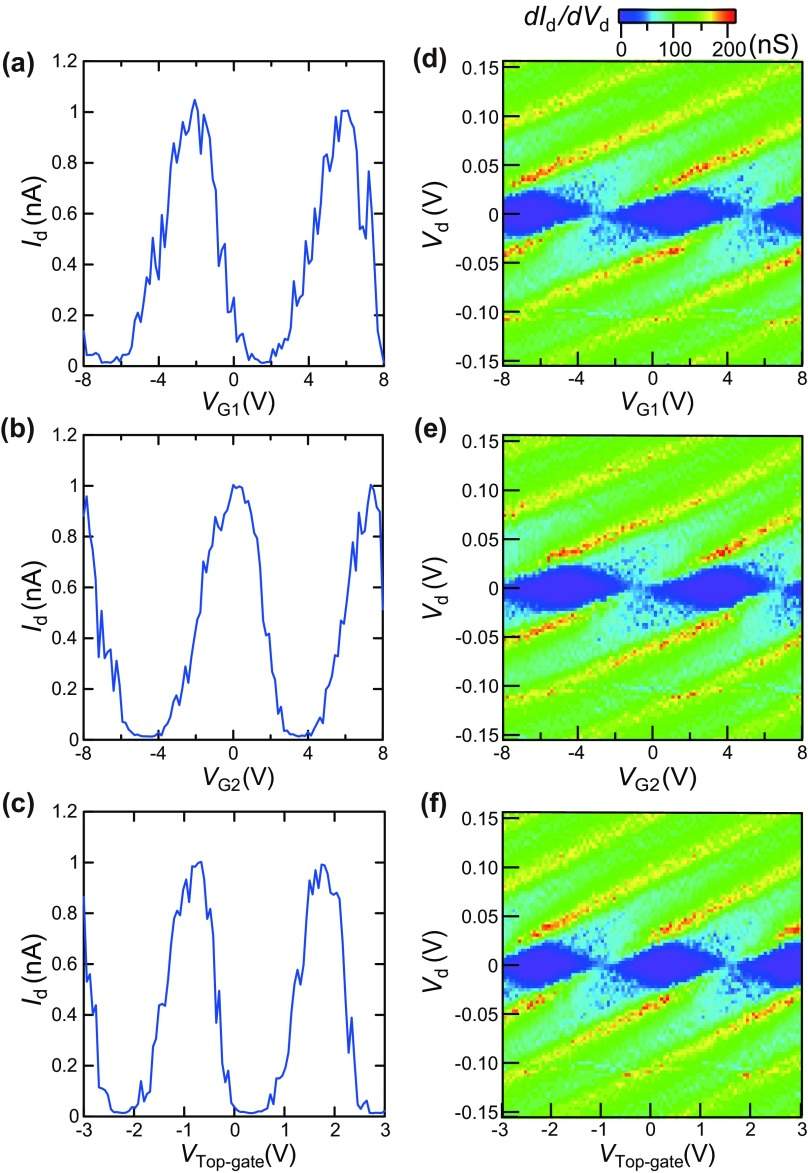
Experimental Coulomb oscillations under the application of (a) side-gate 1 voltage $V_{G}1$, $V_{G}2$=$V_{t}op-gate$=0 V, (b) side-gate 2 voltage $V_{G}2$, $V_{G}1$=$V_{t}op-gate$=0 V, and (c) top-gate voltage $V_{t}op-gate$, $V_{G}1$=$V_{G}2$=0 V with a drain bias voltage $V_{d}$ of 15 mV at 9 K. Experimental stability diagrams under the application of (d) side-gate 1 voltage $V_{G}1$, $V_{G}2$=$V_{t}op-gate$=0 V, (e) side-gate 2 voltage $V_{G}2$, $V_{G}1$=$V_{t}op-gate$=0 V, and (f) top-gate voltage $V_{t}op-gate$, $V_{G}1$=$V_{G}2$=0 V at 9 K.

We then studied the drain current vs. gate voltages characteristics for each gate by applying a bias on gate 1 ($V_{G}1$), gate 2 ($V_{G}2$), and the top-gate ($V_{t}op-gate$). Coulomb oscillations were clearly observed for the side-gates 1 and 2 and for the top-gate, as shown in Figure 3(a)–(c) respectively. In each case, a drain voltage $V_{d}$ of 15 mV was chosen to simplify the comparison. When the peak voltage periods ΔV of the Coulomb oscillations are 8.0, 7.4, and 2.5 V for $V_{G}1$, $V_{G}2$, and $V_{t}op-gate$, the gate capacitances of $C_{G}1$, $C_{G}2$-pagination

and $C_{t}op-gate$ were evaluated by e/ΔV (*e*: unit charge) as 20, 22, and 63 zF, respectively. $C_{G}1$ is nearly identical to $C_{G}2$ and $C_{t}op-gate$ is three times larger than $C_{G}1$ and $C_{G}2$. The numeric values of $C_{G}1$, $C_{G}2$ and $C_{t}op-gate$ are large enough to achieve logic operation under bias voltages of up to 8 V for the three-input gates. It should be noted that the three gate capacitances can be adjusted to nearly identical values by adjusting the thickness of the passivation layer.

**Figure 4.**
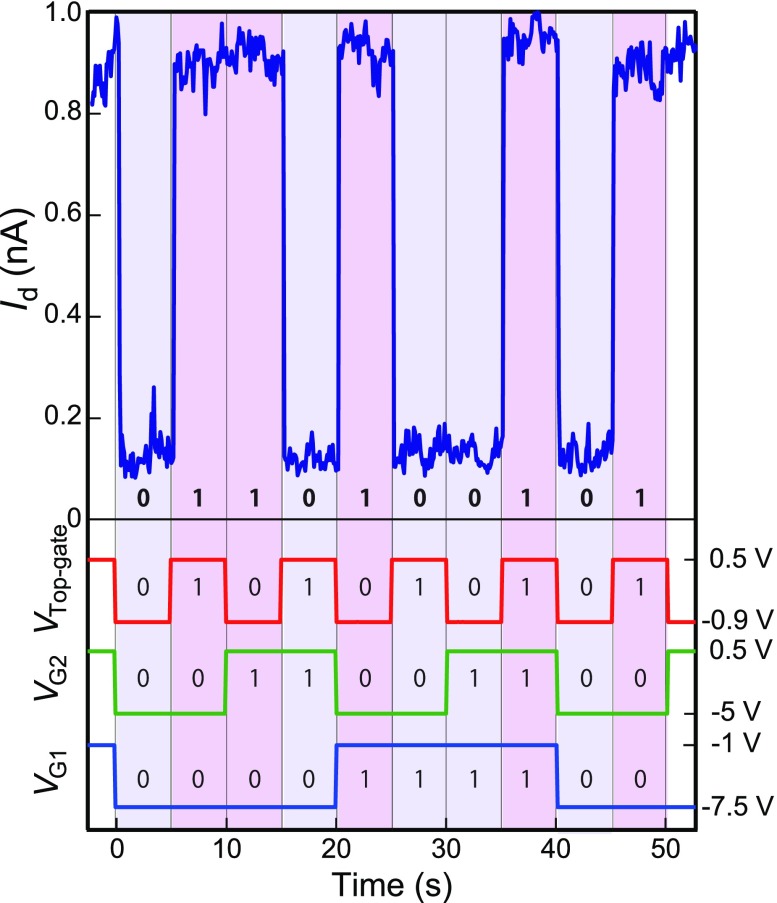
Experimental three-input gate exclusive OR (XOR) logic operation obtained with hybrid SAM and AlO${}_{x}$-passivated chemically assembled SETs. $I_{d}$ output current plot vs. time (blue solid line) under pulse voltages for the top-gate (red), side-gate 1 (blue) and second side-gate 2 (green). The height of the input pulse voltage for side-gate 1 is –7.5 V to –1 V, for side-gate 2 is –5 V to 0.5 V, and for the top-gate is –0.9 V to 0.5 V. Input-output table for three-gate XOR logic operation (inset).

We have previously reported two-input gate logic operation based on the SET without passivation materials and the values of two side-gate capacitances were 28 and 38 zF, respectively [31]. Larger side-gate capacitances are strongly expected by the high-k passivation material of with AlO${}_{x}$; however, the values of $C_{G}1$-pagination (= 20 zF) and $C_{G}2$ (=22 zF) are almost comparable with the previous results [31]. As shown in the SEM image in Figure 1(a), the width of the source and the drain electrodes at the gap is almost 70 nm and the gap separation is 3 nm. Consequently, the electric flux from the side-gate electrodes to Au nanoparticle (6.2 nm in core diameter) tends to be shielded by source and drain electrodes. Conversely, the gap separation of the previous two-input gate SET was as large as 7 nm and the hemispherical source and drain electrode structures were observed at the gap [31]. As a result, larger side-gate capacitances are expected on three-input gates SETs with the hybrid passivation bilayer by extending the gap separation and by reducing the width of the source and the drain electrodes, especially at the gap.

The stability diagrams for each gate (gate 1, gate 2, and top-gate) of the device were then measured at 9 K, as shown in Figure 3(d)–(f), respectively. The uniform Coulomb diamond shape is clearly observed for the three gate voltages in Figure 3(d)–(f). Consequently, the offset charges of the Coulomb islands equally oscillate from gate 1, gate 2, and the top-gate electrodes based on their gate capacitances.

There are 90 SETs on the one sample. After the introduction of the hybrid passivation materials and the top-gate electrode, stable Coulomb diamonds were observed in 13 out of 90 SETs. Within the 13 SETs, five SETs have a single Coulomb island of an Au nanoparticle, which is judged by periodical and uniform Coulomb diamond shape. All experimental results of this paper originate from the same SET with the comparable two side-gate capacitances, which were selected from the five SETs.

The charging energy of this SET is given as $E_{C}=e^{2}/2C_{\Sigma }$, where $C_{\Sigma }=\left(C_{1}+C_{2}+C_{G}1+C_{G}2+C_{t}op-gate\right)$, and is evaluated as 14 meV. We have examined the dependence of $E_{C}$ on the Au core diameter in chemically assembled SETs by introducing a concentric sphere model, and the typical $E_{C}$ for a core diameter of 6.2 nm is 38 meV [22,48]. An $E_{C}$ value of 14 meV in the hybrid SAM and AlO${}_{x}$ passivated chemically assembled SET is nearly three times smaller than that in the chemically assembled SET without the hybrid passivation bilayer. This decrease in $E_{C}$ likely results from the PLD AlO${}_{x}$ passivation material, having a relative permittivity of about 8 [49]. Considering the relative permittivity of alkanethiol protecting molecules (2.6 [50]), the passivation material AlO${}_{x}$ should cover and fill the surrounding space of both the Coulomb island of alkanethiol protected Au nanoparticles and the nanogap regions.

We have previously reported the uniform charging energy of the SETs without the AlO${}_{x}$ passivation material [22]. It can be noted that the uniform charging energy of 14 meV was also observed on the five SETs originating from a single Au nanoparticle even after the introduction of the hybrid passivation bilayer and the top-gate electrodes.

The measurement sequence of Coulomb oscillation and Coulomb diamonds was top-gate, gate 1, and gate 2. Within the Coulomb oscillation and Coulomb diamonds for gate 2 (Figure 3(b) and (e)), the current peak voltage moved to the right compared with those for top-gate and gate 1. This peak shift value is attributed to the change in $Q_{0}$, whose value is evaluated as 0.3*e*. As shown in Figure 3(d) and (f), the stable Coulomb diamonds were clearly observed, which means that $Q_{0}$ did not change during those measurements. As a result, the change in $Q_{0}$ happened during the measurements interval between gate 1 (Figure 3(d)) and gate 2 (Figure 3(e)). It is noted that $Q_{0}$ kept the constant value after this change throughout the measurement of the three gate pulse voltage as follows.

The results of XOR logic operation with three-input gate modulation on the same SET using SAM and AlO${}_{x}$ as the hybrid passivation bilayer are shown in Figure 4(a) and (b). Figure 4(a) shows the output current under the application of the three gate pulse voltages and a drain voltage of 25 mV. Figure 4(b) shows the experimental pulse sequence of three-input gate voltages vs. time with the table of the three-input XOR gate, in which ‘1’ refers to the ON state with current flow and ‘0’ is the OFF state with low current. For side-gate 1 (blue solid line), side-gate 2 (green line), and top-gate (red line), the OFF states are –7.5, –5.0 and –0.9 V and the ON states are –1, 0.5, and 0.5 V, respectively. Depending on the three-input gate voltages, the output current varied between 0.9 and 0.12 nA, which are defined as ON and OFF states (Figure 4(a)). Times between 0 and 5 s (input state: 0, 0, and 0) correspond to the beginning of the output in the table (output state: 0). We obtained the correct XOR logic output for each input step, as the ON state of the output current appears only when the sum of the ON state inputs is an odd number.

The ON/OFF ratio of three-input XOR logic is 7.5, which is almost one order of magnitude in difference. The output ON and OFF current are approximately 900 and 120 pA, respectively. We have previously reported two-input chemically assembled SET logic circuit operation in which the ON/OFF ratio and OFF current were $10^{2}$ and 0.1 pA, respectively [31]. A large OFF current of 120 pA under $V_{d}$ of 25 mV is due to almost outside of Coulomb blockade region; however, we have only tried to measure three-input gate XOR logic operation under $V_{d}$ of 25 mV. Based on the experimental Coulomb staircases at ON ($Q_{0}$ = 0.5*e*) and OFF ($Q_{0}$ = 0) states in Figure 2, ON and OFF currents under $V_{d}$ of 15 mV were 900 and 0.7 pA, respectively. Consequently, ON/OFF ratio of $10^{3}$ was expected by choosing $V_{d}$ of 15 mV.

Due to the small side-gate capacitances of 20 and 22 zF, the gate voltages for the logic operation were slightly large, e.g. –5 V and –7.5 V. We are now trying to reduce the width of the source and drain electrodes at the gap comparable for the size of an Au nanoparticle, which will lead the lower-voltage operation by a geometry improvement.

## 4. Conclusions

We have demonstrated three-input gate XOR logic operation on chemically assembled SETs by introducing a hybrid SAM and AlO${}_{x}$ passivation bilayer. Based on the small charging energy and the large leakage current, the high-k material of a PLD AlO${}_{x}$ was found to fill the space surrounding the Au nanoparticles and the nanogap electrodes. Three-input XOR logic operation was stable and reproducible with an ON/OFF ratio of 7.5. This hybrid SAM and AlO${}_{x}$ passivation is significant for the fabrication of sub-10-nm chemically assembled SETs to function as logic devices, since it enables connection to CMOS technologies via planar processes.
